# 

**Published:** 1853-04

**Authors:** 


Vol. VI. APRIL, 1853. No. 3.

THE

### DENTAL REGISTER

OF

### THE WEST.

EDITED BY

JAMES TAYLOR, M. D., D. D. S.

PUBLISHED QUARTERLY.
AT $2 PER ANNUM.

AGENTS.

J. B. Dunlevy, Pittsburgh, Pa.
Drs. Spalding & Fithian, St. Louis, Mo.
Messrs. Jones, White & McCurdy, Phila. New York City. and Boston, Mass.
Dr. J. M. Brown, Cincinnati.
Solomon Brown, New York city.
John Klein, Phil. Pa.
Dr. Edward McCuen, Traveling Agent.

CINCINNATI:
PRINTED BY T. WRIGHTSON, 12 WEST SECOND STREET.
1853.

